# Editorial: Migration and health: a human rights perspective - conference insights and beyond

**DOI:** 10.3389/fpubh.2025.1733468

**Published:** 2025-11-14

**Authors:** Noemí Rodríguez, Mira Goral, Justine McGovern, Allison Squires, María Isabel Roldós

**Affiliations:** 1 New York City College of Technology, City University of New York (CUNY), Brooklyn, NY, United States; 2 CUNY Institute of Health Equity, Bronx, NY, United States; 3 Lehman College, City University of New York (CUNY), Bronx, NY, United States; 4 Global Consortium of Nursing and Midwifery Studies, Rory Meyers College of Nursing, New York University, New York, NY, United States

**Keywords:** migration, migrant, health, disparity, human rights, mobility

Human mobility is a world-wide phenomenon among global majority and global minority countries (9). The number of international migrants has been growing; it was about 300 million in 2024 (1, 8). People move for a variety of reasons, such as seeking better jobs and access to better health care; running from famine, war, or natural disasters; and fleeing persecution (2). Regardless of the rationale, people who move are likely to experience health disparities–characterized by limited access to care; cultural, language, and social barriers; and adverse physical and mental health outcomes (3).

To raise awareness and ignite collaborations to strategically mitigate health disparities prompted by transitions from home countries to new environments, the City University of New York Institute for Health Equity (CIHE) led a 2023 Academic Summit on “*Migration and Health: A Human Rights Perspective*.” Invigorated by the summit's timely and important dialogues, we sought to widen the discussion and scholarly contributions–ensuing this Research Topic.

With a stimulating compilation of 11 articles, the Research Topic includes quantitative and qualitative studies, reviews of current knowledge with future outlooks, and commentaries embedded in existential research to challenge the status quo and act toward health equity among populations experiencing mobility. Combined, the scholarship conveys the cycle of migration through the lifespan (from maternal and infant care to older care) and addresses a variety of morbidities (physical and mental) as well as potential actions and solutions (patient-centered approaches to care, robust education and social support programs).

Examining health disparity across the lifespan, six articles provide insight from maternal care (impacting fetal care) to older care. Focused on maternal care, Smith et al.'s interviews with South Asian refugees living in the US highlight the flaws of the US healthcare system as they are amplified for refugees, whose unfamiliarity with the health system of the new country limits care. Recommendations include investments in nursing care, multilingual doula care, language services, and robust childbirth education. This is most relevant given high maternal and infant mortality rates in the US (4). Lessons from the US can be presented as case studies for other peer countries.

Focusing on child health, Jeong approaches healthcare access and migration from the perspective of structural barriers induced by strict government regulations. Through a content analysis of documents spanning 17 years, Jeong identifies China's denial of legal identity among children of North Korean defectors as a key limiting factor to access pediatric care. To improve the situation, Jeong recommends advocating for policies that dissociate the legality of a child from health services. Maintaining a focus on young migrants and policy, Vega examines undocumented and immigrant students experiences with distress and explores solutions that can help mitigate negative consequences of immigration policies. The author introduces the Immigration Battle Fatigue model and discusses pathways for supporting mental health of student migrants. Similarly focusing on students in higher education institutions, Reynoso et al. conducted a peer-to-peer interview study with students from diverse backgrounds to examine how STEM studies are perceived. The authors identify key themes, including the importance of time management support, diverse mentors, and breaking down stereotypes. As in other papers in the Research Topic, the critical role of authentic support emerged.

Moving toward wellbeing at the later end of the lifespan, Zhou et al. interviewed older individuals from diverse linguistic and cultural backgrounds living in Sweden. Their findings (1) point to a desire to understand and be understood, emphasizing the critical role of communication and education and (2) highlight the resilience that the participants demonstrated through identity negotiation and cultural adaptation strategies. The authors conclude that person-centered approaches in care–sensitive to cultural differences–have the potential to support immigrants despite complex psychosocial processes and communication barriers. With a continued focus on the older adult(s), Muñoz-Alicea and Suarez-Gomez qualitatively examine depression among informal caregivers of older Hispanic adults residing in Mexico, Puerto Rico, and Columbia. The investigation reveals aspects that affect mental health among caregivers of older Hispanics and highlights the importance of social support. Focusing on caregivers for older adults is innovative. Moreover, older care among migrants is important given the growing aging population worldwide (5).

Together with the work by Jeong and by Vega mentioned above, two additional articles focus on systems, structures, and the social ecology of societies. Martens et al. examine migrant status and access to vaccination during the COVID19 pandemic using survey data from Ecuador. Amid barriers and gaps in access, solutions emerged, including support from non-governmental organizations, mobile health brigades, and pressure from international organizations. Education and communication also play a role in improving access to care among refugees and migrants.

The critical role of understanding cultural diversity and the importance of moving away from a colonial view of healthcare are emphasized in McGovern and Fusco's review paper. The review focuses on epistemic disobedience in public health research and practice in the context of migration. The authors define and situate terms in historical and current literature while critically exploring the usage of epistemic disobedience in public health and migration scholarship. The review also points to promising future applications of epistemic disobedience and implications in public health and migration research and practice.

Three articles attend to health areas often passed over: mental health and dental care. Mental health care is discussed in Blukacz et al. The authors examine self-reported stress and mood disorders in relation to demographic, socioeconomic, health, and migration-related factors among international migrants in Chile. Data from a structured questionnaire administered to 1,656 international migrants and 1,664 locals revealed worse mental health indicators for the migrants (e.g., higher rates of mood disorders) compared to the locals; these differences did not change with length of residence. Nevertheless, acculturation processes may offer potential for decreasing mental health challenges among immigrants.

Much like mental health, oral health is often overlooked. Martín Hernández et al.'s cross-sectional study with 128 adult migrants residing in a temporary center in Spain analyzes the association between oral health status and social determinants of health. They concluded that this population experienced major oral health challenges and that disparities were evident by a migrant's home country, education, and ability to access care. Further monitoring is needed, especially since oral health is closely related to systemic health (6, 7).

Finally, Izquierdo-Condoy et al. examine the wellbeing of a special group of migrants–those who are forced to return to their original countries. The authors discuss challenges facing returnees, including limited healthcare access, economic instability, and social exclusion; and emphasize how these barriers contribute to widening health disparities. They suggest that systematic, global efforts are needed to mitigate the negative consequences of these marginalized groups.

The strength of this Research Topic lies in its breadth of methodological approaches, variety of populations spanning the human lifespan, countries of the globe, health aspects addressed, and global perspectives. It brings together scholars from 12 countries and one US territory, focusing on distinct migrant groups and a variety of potential action plans for improving health outcomes and minimizing health disparity.
