# Correction: Editorial: Migration and health: a human rights perspective - conference insights and beyond

**DOI:** 10.3389/fpubh.2025.1751697

**Published:** 2025-12-08

**Authors:** Noemí Rodríguez, Mira Goral, Justine McGovern, Allison Squires, María Isabel Roldós

**Affiliations:** 1 New York City College of Technology, City University of New York (CUNY), Brooklyn, NY, United States; 2 CUNY Institute of Health Equity, Bronx, NY, United States; 3 Lehman College, City University of New York (CUNY), Bronx, NY, United States; 4 Global Consortium of Nursing and Midwifery Studies, Rory Meyers College of Nursing, New York University, New York, NY, United States

**Keywords:** migration, migrant, health, disparity, human rights, mobility

Authors Justine McGovern, Allison Squires, and María Isabel Roldós were erroneously assigned the symbol (†) noting equal contributing to all authors.

The correct citation is:

Noemí Rodríguez^1, 2†^, Mira Goral^2, 3†^, Justine McGovern^2, 3^, Allison Squires^4^, María Isabel Roldós^2, 3*^

The original version of this article has been updated.

